# *Ept7*, a quantitative trait locus that controls estrogen-induced pituitary lactotroph hyperplasia in rat, is orthologous to a locus in humans that has been associated with numerous cancer types and common diseases

**DOI:** 10.1371/journal.pone.0204727

**Published:** 2018-09-27

**Authors:** Kirsten L. Dennison, Aaron C. Chack, Maureen Peters Hickman, Quincy Eckert Harenda, James D. Shull

**Affiliations:** 1 McArdle Laboratory for Cancer Research, Department of Oncology, School of Medicine and Public Health, University of Wisconsin, Madison, Wisconsin, United States of America; 2 University of Wisconsin Carbone Cancer Center, School of Medicine and Public Health, University of Wisconsin, Madison, Wisconsin, United States of America; Medical College of Wisconsin, UNITED STATES

## Abstract

Pituitary adenoma is a common intracranial neoplasm that is observed in approximately 10% of unselected individuals at autopsy. Prolactin-producing adenomas, i.e., prolactinomas, comprise approximately 50% of all pituitary adenomas and represent the most common class of pituitary tumor. Multiple observations suggest that estrogens may contribute to development of prolactinoma; however, direct evidence for a causal role of estrogens in prolactinoma etiology is lacking. Rat models of estrogen-induced prolactinoma have been utilized extensively to identify the factors, pathways and processes that are involved in pituitary tumor development. The objective of this study was to localize to high resolution *Ept7* (*E*strogen-induced *p*ituitary *t*umor), a quantitative trait locus (QTL) that controls lactotroph responsiveness to estrogens and was mapped to rat chromosome 7 (RNO7) in an intercross between BN and ACI rats. Data presented and discussed herein localize the *Ept7* causal variant(s) to a 1.91 Mb interval of RNO7 that contains two protein coding genes, *A1bg* and *Myc*, and *Pvt1*, which yields multiple non-protein coding transcripts of unknown function. The *Ept7* orthologous region in humans is located at 8q24.21 and has been linked in genome wide association studies to risk of 8 distinct epithelial cancers, including breast, ovarian, and endometrial cancers; 3 distinct types of B cell lymphoma; multiple inflammatory and autoimmune diseases; and orofacial cleft defects. In addition, the *Ept7* locus in humans has been associated with variation in normal hematologic and development phenotypes, including height. Functional characterization of *Ept7* should ultimately enhance our understanding of the genetic etiology of prolactinoma and these other diseases.

## Introduction

Pituitary adenoma is a common intracranial neoplasm that is observed in approximately 10% of unselected individuals examined at autopsy [1–3]. The etiology of pituitary adenoma is poorly understood. Mutations in a small number of genes are known to underlie distinct genetic syndromes in which pituitary adenoma is observed. Examples of pituitary adenoma associated genes and syndromes include: *MEN1*, multiple endocrine neoplasia type I; *CDKN1B*, multiple endocrine neoplasia type IV; and *PRKAR1A*, Carney complex type 1 [4]. In addition, non-syndromic forms of familial pituitary adenoma are becoming recognized. One such class of familial isolated pituitary adenoma results from mutations in *AIP* [5]. By contrast, the majority of pituitary adenomas appear to be sporadic in origin, suggesting that acquired somatic mutations and/or common germline variants acting with low penetrance may contribute to development of these tumors.

Prolactin-producing adenomas, i.e., prolactinomas, comprise approximately 50% of all pituitary adenomas and represent the most common class of pituitary tumor [1, 6–8]. The incidence of prolactinoma is approximately 10-fold higher in females of childbearing age than in postmenopausal women or men [8]. In addition, multiple case reports have described development of prolactinoma in male to female transsexuals who undergo treatment with estrogens to induce female secondary sex traits [9–17]. Together, these observations suggest that estrogens contribute to the development of prolactinoma, but direct evidence for a causal role of estrogens in prolactinoma etiology in humans is lacking. Although prolactinoma can be locally invasive, these tumors rarely progress to malignant carcinoma. Nonetheless, prolactinoma frequently results in multiple endocrinological and/or neurological pathologies. Prolactinoma is often causally associated with hyperprolactinemia, which in turn results in disruption of the normal pattern of secretion of gonadotropins, reduced production of gonadal steroids, amenorrhea, and infertility in females, as well as decreased libido and infertility in males [4, 7]. The expansion of the lactotroph population within the anterior pituitary gland can also lead to dysregulation of other pituitary secretory cell types, thereby resulting in other endocrine pathologies. Moreover, physical expansion of the tumor mass can result in compression of the optic chiasm and other vital anatomical structures, leading to impaired vision, headache and/or other neurological problems.

Rat models of estrogen-induced prolactinoma have been utilized extensively to identify the factors, pathways and processes that are involved in pituitary tumor development [18–21]. It has long been recognized that the proliferative response of the pituitary lactotroph population to estrogens differs dramatically between different rat strains [22–26]. Fischer 344 (F344) and August x Copenhagen, Irish (ACI) are two inbred rat strains that are highly responsive to estrogens, whereas the outbred Sprague-Dawley and inbred Brown Norway (BN) strains are restrained in their responsiveness [20]. This inter-strain variation in lactotroph responsiveness to estrogens has been exploited in genetic studies to advance understanding of the mechanisms through which estrogens regulate lactotroph proliferation and/or survival and contribute to development of prolactinoma [27–36]. The objective of this study was to fine map the location of *Ept7* (*E*strogen-induced *p*ituitary *t*umor), a quantitative trait locus (QTL) that controls lactotroph responsiveness to estrogens and was mapped to rat chromosome 7 (RNO7) in an intercross between BN and ACI rats [34]. The existence of *Ept7* was confirmed by generation and characterization of a congenic rat strain that harbors BN alleles across the *Ept7* interval on the ACI genetic background [36]. Data presented and discussed in this manuscript localize the *Ept7* causal variant to a 1.91 Mb interval of RNO7 that contains only two protein coding genes and one or more genes that yield non-protein coding transcripts of unknown function. *Ept7* is orthologous to an interval within the 8q24.21 region of the human genome that has been associated with risk of numerous cancer types and other common diseases. These data may ultimately enhance our understanding of the genetic etiology of prolactinoma in humans.

## Materials and methods

### Care, treatment, and phenotypic characterization of animals

All procedures involving live animals were approved by the Institutional Animal Care and Use Committee of the University of Wisconsin-Madison (protocol #M005111). ACI/SegHsd (ACI) and BN/SsNHsd (BN) rats were obtained from Envigo (formerly Harlan Sprague Dawley, Inc.). Each of the unique congenic rat strains described herein was generated in our laboratory as described below. All rats were housed under controlled temperature, humidity, and 12-hr light/12-hr dark conditions in facilities that were accredited by the American Association for Accreditation of Laboratory Animal Care and operated in accordance with *The Guide for the Care and Use of Laboratory Animals* [37].

Female rats from each rat strain were treated with 17β-estradiol (E2) beginning at 9 weeks of age as described previously [38–42]. Control rats from each strain received empty implants. Implants, empty or containing 27.5 mg of E2 (Sigma-Aldrich, St. Louis, MO), were prepared from Silastic tubing (Dow Corning, Midland, MI) and sealed with Silastic Medical Adhesive (Silicone Type A; Dow Corning). Implants were placed surgically into the interscapular region of 9 week old rats. During the procedure, rats were anesthetized with isoflurane administered at a rate of 1–5% via inhalation using an anesthetic vaporizer. Because the studies described herein were performed over a five year interval, fifteen groups of ACI females, totaling 77 animals, were evaluated starting at different points across this interval to ensure that adequate numbers of ACI rats were treated contemporaneously with rats from each of the different congenic strains. The pituitary weight phenotype in the ACI rats was stable over the five year interval (S1 Fig). Therefore, data from the different groups of ACI rats were pooled for statistical comparison to rats from the different congenic strains. Research staff monitored the rats a minimum of three times per week and weighed the rats weekly. The rats were generally euthanized following 196 ± 5 days of treatment. However, an animal was euthanized prior to the intended experiment endpoint if it exhibited cachexia (loss of 15% body mass); poor body condition; loss of normal neuromuscular coordination; abdominal swelling; or a mammary tumor that grew in excess of 2 cm in longest dimension, became ulcerated or impeded normal ambulation. The pituitary gland was collected at necropsy, weighed, and photographed. Pituitary weight has been shown to correlate with pituitary DNA content and the level of circulating prolactin, and thereby serves as a surrogate indicator of absolute lactotroph number [20, 29, 35, 36, 38].

### Generation of congenic rat strains

Each of the congenic strains was developed using a marker assisted selective breeding protocol as previously described [36, 39]. When a male was obtained that was heterozygous for BN alleles across a desired segment of RNO7 and homozygous for ACI alleles at all background markers, that male was backcrossed to ACI females and sibling progeny carrying the same recombinant chromosome were intercrossed to produce rats homozygous for BN alleles across the locus of interest. The different congenic strains are referred to by their abbreviated names: Ept7.1 through Ept 7.18. The full strain names and Rat Genome Database (RGD) identification numbers are listed in S1 Table. Genome coordinates for the BN intervals on RNO7 carried by each congenic strain are listed in S1 Table.

### Statistical analyses of data

Phenotypes exhibited by Ept7 congenic rats were compared to those of treated ACI rats. Pituitary weights and treatment duration were expressed as the mean ± standard error of the mean (SEM). Differences in pituitary weight relative to ACI were evaluated using the Wilcoxon Rank Sum test. The impact on survival of morbidities related to pituitary hyperplasia/adenoma was evaluated using the Kaplan-Meier estimator and log rank test. Animals that were removed from the study due to mammary tumor burden or any mammary cancer associated morbidity were censored in these analyses. The thresholds for statistical significance were adjusted for multiple comparisons using the Bonferroni method. MSTAT Version 6.1 was used to perform all statistical analyses [43].

### Sources of microarray data

Gene expression data for *Ept7* candidates were generated using Affymetrix Rat Genome 230 version 2.0 GeneChips as described previously [40]. The biological samples from sham treated and diethylstilbestrol (DES) treated male ACI and BN rats were part of a larger study focused on genetic control of estrogen action in the anterior pituitary gland and the resulting data were deposited in the GEO Database under accession number GSE4028.

### Sources of genomic data

Rat and human genome coordinates in this manuscript refer to genome assemblies Rnor_6.0 and GRCh38.p10, respectively. All genetic markers, marker locations, and rat strains are registered in the Rat Genome Database [44, 45]. Genomic features of the *Ept7* locus are from Ensembl Rnor_6.0 release 91 [46].

## Results

### Phenotypic characterization of 19 unique ACI.BN-Ept7 congenic rat strains

*Ept7* was previously mapped to a 32.4 Mb interval on RNO7 defined by microsatellite markers *D7Wox3* (71.45 Mb) and *D7Rat17* (103.85 Mb) [34, 36]. *Emca4* (*E*strogen-induced *m*ammary *ca*ncer), a QTL that influences development of E2-induced mammary cancer, was mapped to this same region of RNO7 in a BN x ACI intercross [42]. In order to further localize the *Ept7* and *Emca4* causal variants we generated and characterized multiple congenic rat strains, each of which is homozygous for BN alleles across a unique segment of RNO7 introgressed onto the ACI genetic background (S1 Table). The data presented here are focused on the mapping of *Ept7*. Data relating to the mapping of *Emca4* will be published elsewhere and are discussed only when relevant to *Ept7*.

Pituitary weight, a surrogate indicator of absolute lactotroph number, was measured as the primary phenotypic indicator of lactotroph responsiveness to E2 [20, 29, 35, 36, 38]. Pituitary weight for sham treated control rats across all 21 rat strains examined in this study averaged 9.8 mg (standard error of the mean = 0.2; n = 105). No significant differences were observed when basal pituitary weight for BN rats or rats from each of the 19 unique congenic strains was compared to basal pituitary weight for ACI rats, which served as the background (recipient) strain during the generation of the congenic rat strains characterized in this study (Table 1).

**Table 1:** Pituitary hyperplasia phenotypes.

	Sham treatment						E2-treatment					
Strain	Pituitary Weight ^*a*^, mg	P vs ACI ^*b*^	Days Treated ^*a*^	Survival ^*c*^, %	P vs ACI ^*d*^	N ^*e*^	Pituitary Weight ^*a*^, mg	P vs ACI ^*b*^	Days Treated ^*a*^	Survival ^*c*^, %	P vs ACI ^*d*^	N ^*e*^
ACI ^*f*^	10.5 ± 0.7	-	194.6 ± 0.1	100.0	-	12 (12)	189.9 ± 8.0	-	185.4 ± 1.7	51.5	-	70 (77)
BN	8.5 ± 0.8	0.0818	195.0 ± 0	100.0	1	5 (5)	16.2 ± 0.7	1.23e-08	196.4 ± 0.2	100.0	0.0159	9 (10)
Ept7	10.8 ± 0.8	0.9593	196.4 ± 0.4	100.0	1	5 (5)	104.7 ± 8.9	8.84e-08	192.2 ± 2.1	91.8	0.0012	23 (26)
Ept7.1	7.7 ± 0.9	0.0703	197.0 ± 0	100.0	1	3 (3)	166.1 ± 13.9	0.1647	188.3 ± 3.2	59.7	0.3510	27 (29)
Ept7.2	9.1 ± 0.8	0.1902	196.0 ± 0	100.0	1	4 (4)	139.3 ± 8.7	0.0004	188.6 ± 2.8	72.0	0.0578	28 (27)
Ept7.3	9.1 ± 0.8	0.2358	196.0 ± 0	100.0	1	4 (4)	134.9 ± 11.6	0.0008	189.2 ± 3.0	81.5	0.0127	24 (25)
Ept7.4	11.9 ± 1.6	0.5743	196.0 ± 0	100.0	1	5 (5)	175.3 ± 11.6	0.2844	186.9 ± 2.4	43.4	0.4499	27 (31)
Ept7.5	9.1 ± 0.7	0.2133	196.0 ± 0	100.0	1	5 (5)	178.2 ± 13.8	0.5760	181.0 ± 4.0	37.4	0.3926	22 (26)
Ept7.6	8.1 ± 0.7	0.1036	196.0 ± 0	100.0	1	5 (5)	120.3 ± 9.0	2.89e-06	190.1 ± 2.4	95.2	0.0005	26 (26)
Ept7.7	11.8 ± 0.4	0.2343	196.0 ± 0	100.0	1	5 (5)	166.1 ± 12.8	0.0845	185.9 ± 3.4	64.1	0.2273	22 (24)
Ept7.8	10.7 ± 0.5	0.9594	197.0 ± 0	100.0	1	5 (5)	200.3 ± 14.6	0.5038	177.1 ± 5.0	31.9	0.1456	22 (24)
Ept7.9	11.7 ± 1.2	0.6097	196.0 ± 0	100.0	1	5 (5)	236.4 ± 16.0	0.0215	176.6 ± 3.7	15.2	0.0018	20 (24)
Ept7.10	8.8 ± 0.6	0.1698	196.0 ± 0	100.0	1	4 (5)	116.1 ± 12.3	2.32e-05	189.3 ± 3.2	84.2	0.0102	20 (26)
Ept7.11	8.7 ± 0.6	0.1296	198.0 ± 1.2	100.0	1	5 (5)	158.3 ± 9.4	0.0221	194.4 ± 0.8	76.9	0.0243	24 (26)
Ept7.12	11.0 ± 0.8	0.6835	196.0 ± 0	100.0	1	4 (4)	181.3 ± 10.6	0.6356	183.1 ± 2.1	25.9	0.1057	24 (24)
Ept7.13	9.3 ± 0.7	0.4412	196.0 ± 0	100.0	1	5 (5)	210.4 ± 12.7	0.2165	186.6 ± 3.7	72.7	0.0862	24 (24)
Ept7.14	8.3 ± 0.6	0.0637	196.0 ± 0	100.0	1	5 (5)	168.2 ± 8.8	0.1413	194.6 ± 0.8	85.7	0.0073	21 (21)
Ept7.15	9.5 ± 0.7	0.3827	196.0 ± 0	100.0	1	5 (5)	212.0 ± 11.2	0.1522	186.0 ± 2.5	59.4	0.5812	24 (28)
Ept7.16	9.6 ± 0.2	0.2786	196.0 ± 0	100.0	1	5 (5)	118.2 ± 12.6	1.65e-05	184.1 ± 3.8	72.7	0.0486	25 (30)
Ept7.17	9.9 ± 0.4	0.4421	196.0 ± 0	100.0	1	5 (5)	141.3 ± 9.0	0.0015	191.9 ± 2.7	95.8	0.0006	23 (24)
Ept7.18	9.7 ± 0.7	0.3791	196.0 ± 0	100.0	1	4 (5)	123.7 ± 8.5	6.22e-06	195.0 ± 0.8	100.0	0.0001	25 (26)

^*a*^ Mean ± standard error of the mean for all rats with pituitary weights recorded at necropsy.

^*b*^ Calculated using Wilcoxon rank-sum test.

^*c*^ Kaplan-Meier survival probability estimate for the population at endpoint, for pituitary hyperplasia-associated morbidity (all other losses censored).

^*d*^ Calculated from morbidity data using log rank test.

^*e*^ All rats with pituitary weights recorded at necropsy (all rats with accurate morbidity data).

^*f*^ Data published previously [38].

Continuous treatment with E2 induced pituitary lactotroph hyperplasia, as evidenced by significant increases in pituitary weight, in females of each of the 21 rat strains examined in this study. For reference, pituitary weight in the ACI strain was increased 18.1-fold, from 10.5 ± 0.7 mg in sham treated (control) female rats to 189.9 ± 8.0 mg in rats treated with E2 (Table 1). By contrast, female rats of the BN strain, which served as the donor strain during generation of the congenic rat strains described in this manuscript, exhibited a 1.9-fold increase in pituitary weight in response to prolonged treatment with E2, from 8.5 ± 0.8 mg in sham treated controls to 16.2 ± 0.7 mg. Evaluation of pituitary weight in E2 treated females from each of the 19 congenic rat strains revealed two distinct groups of strains that differed in responsiveness to E2. Rats from the congenic strains in Group 1 were highly responsive to E2 and exhibited pituitary weights that did not differ from those exhibited by ACI rats (Table 1). Group 1 included the Ept7.1, Ept7.4, Ept7.5, Ept7.7, Ept7.8, Ept7.9, Ept7.12, Ept7.13, and Ept7.15 congenic strains. Rats from the congenic stains in Group 2 exhibited pituitary weights that were significantly less than that exhibited by ACI rats, indicating that the responsiveness of the pituitary lactotroph in these rat strains is reduced relative to that of ACI rats. Group 2 consisted of the Ept7, Ept7.2, Ept7.3, Ept7.6, Ept7.10, Ept7.16, Ept7.17, and Ept7.18 congenic rat strains. The Ept7.11 and Ept7.14 congenic strains were not assigned to either Group 1 or Group 2 for reasons discussed below.

**Fig 1:**
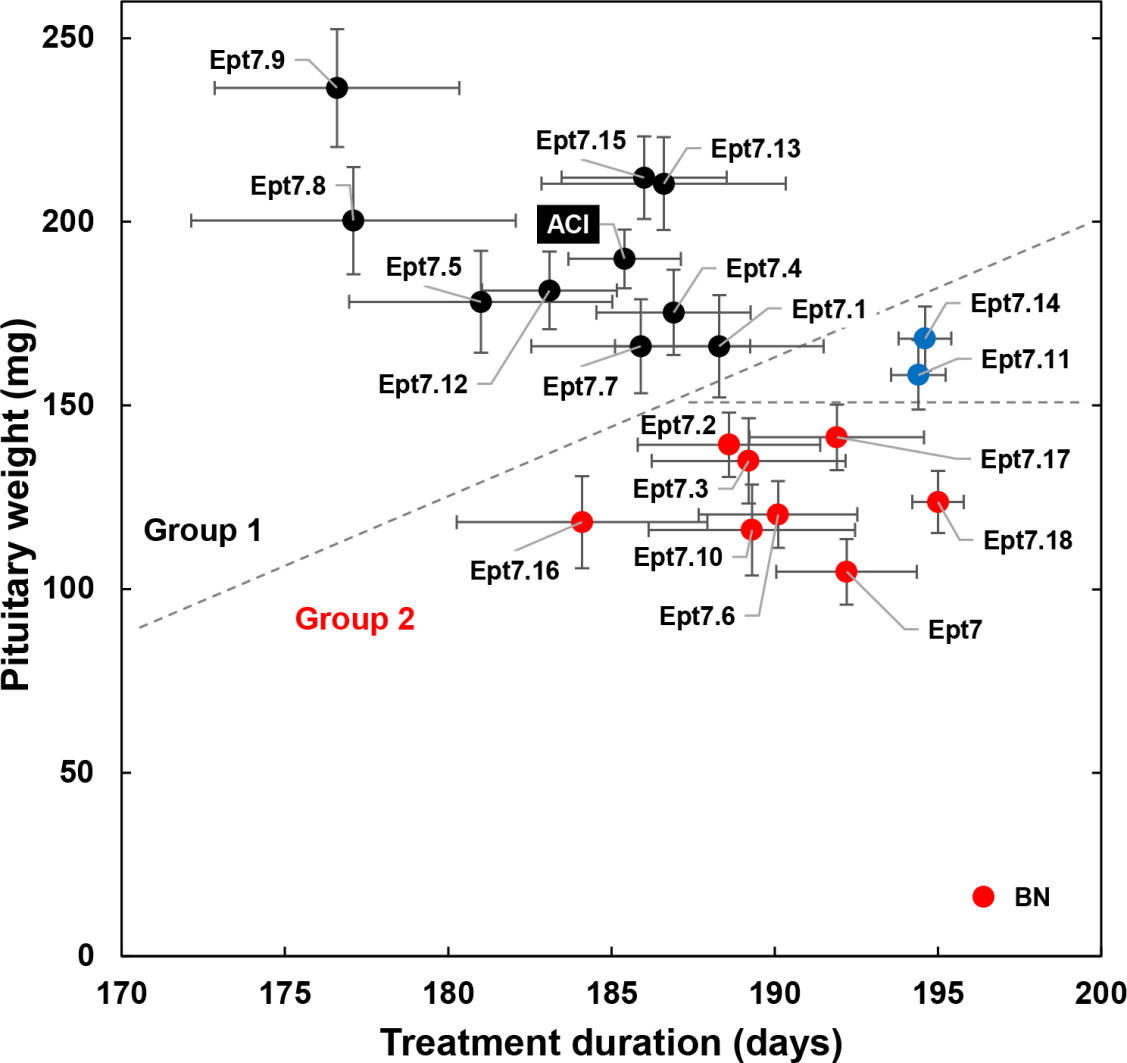
Pituitary weights in ACI, BN and Ept7 congenic rats. Female rats were treated with E2, released from subcutaneous Silastic implants, beginning at 9 weeks of age as described in Materials and Methods. Animals were euthanized upon observation of any treatment associated morbidity or following 28 weeks of treatment. Pituitary weight measured at necropsy is indicated on the y-axis; duration of treatment is indicated on the x-axis. Each data point represents means ± SEMs; N = 9–70 for each strain. Red symbols indicate mean pituitary weights that are significantly less than exhibited by ACI rats (P < 0.0025 by Wilcoxon rank sum test). The dashed lines demarcate those congenic strains that were assigned to Groups 1 (pituitary weights not different from ACI) and Groups 2 (pituitary weights less than ACI). Average pituitary weight in Ept7.11 and Ept7.14 rats (blue symbols) did not differ from that observed in ACI rats; however, the Ept7.11 and Ept7.14 rats were treated with E2 for a longer duration (approximately 9 days) than ACI rats.

Rats were removed from the study upon evidence of morbidity resulting from pituitary hyperplasia/adenoma, mammary cancer, or unknown etiology. Therefore, we plotted pituitary weight for each strain as a function of the duration of E2 treatment (Fig 1). We also evaluated survival of E2 treated rats from each congenic strain as a function of morbidities that are known to result from lactotroph hyperplasia/adenoma and gross pituitary enlargement (Table 1). The resulting data indicate that rats from the Group 2 congenic strains, with the exception of rats from the Ept7.16 strain, were generally treated with E2 for a longer duration than ACI rats. Thus, the inhibitory actions of BN alleles at *Ept7* in the Group 2 congenic strains on E2-induced pituitary growth were likely to have been underestimated due to the longer durations of treatment (discussed further below).

### *Ept7* causal variant resides within a 1.91 Mb interval on RNO7

**Fig 2:**
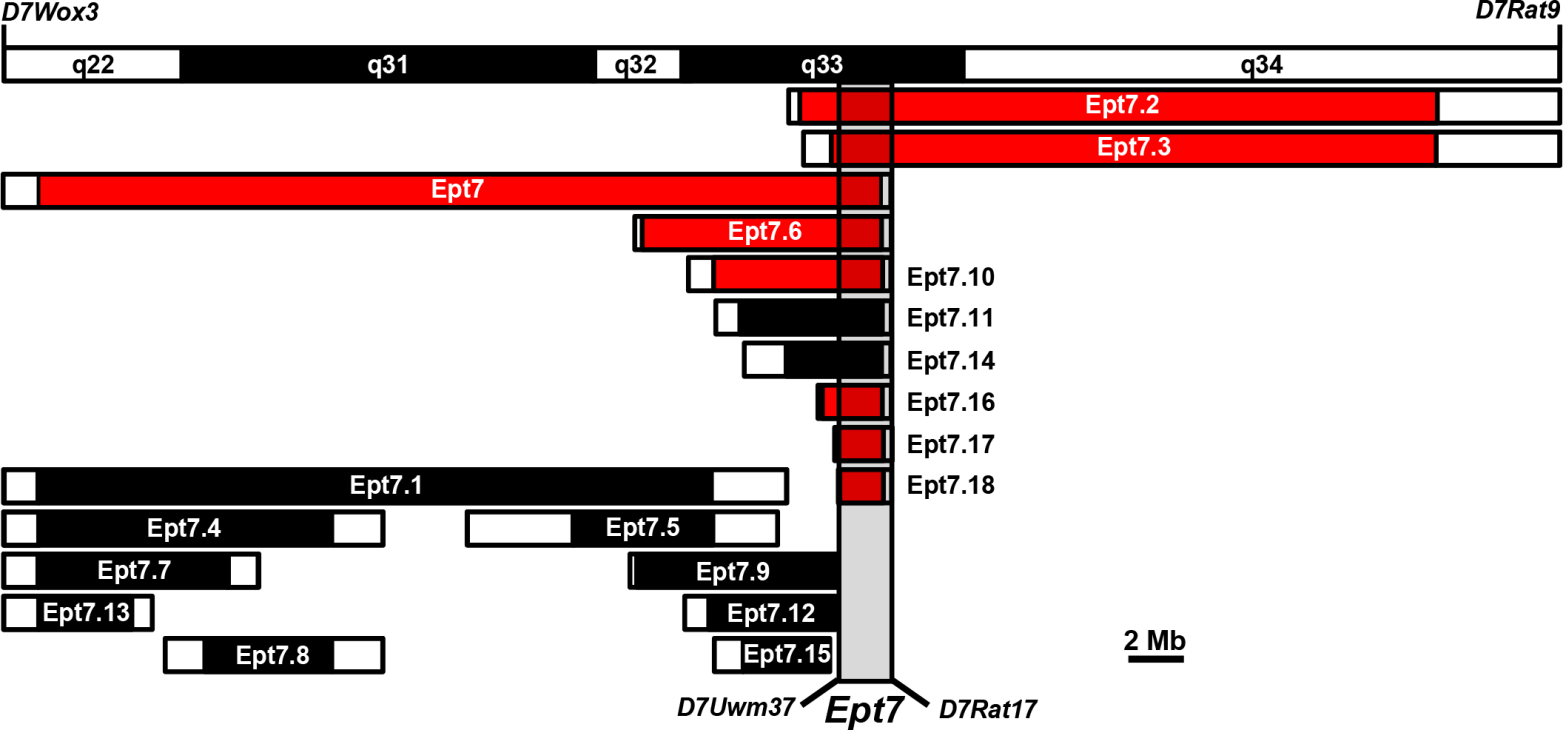
*Ept7* maps to a 1.91 Mb interval on rat chromosome 7. The ideogram at the top of the figure depicts the section of rat chromosome 7 that extends from *D7Wox3* (71.45 Mb) to *D7Rat9* (128.09 Mb). Each black or red filled horizontal box indicates the segment of RNO7 from the donor BN rat strain that was introgressed onto the genetic background of the recipient ACI strain to generate the indicated congenic rat strains. The white segments flanking a black or red box indicate regions of recombination (i.e., unknown genotype); all other segments of RNO7 were derived from the recipient ACI strain. Black boxes indicate congenic intervals in strains that did not exhibit reduced pituitary weight when compared to E2 treated ACI rats, whereas red boxes indicate congenic intervals in strains that exhibited reduced pituitary weight (P < 0.0025 by Wilcoxon rank sum test). The minimal *Ept7* interval extends over the 1.91 Mb segment of RNO7 from *D7Uwm37* (101.94 Mb) to *D7Rat17* (103.85 Mb), illustrated by the gray vertical box.

Examination of the BN genome intervals harbored by each of the congenic rat strains localized the *Ept7* causal variant(s) within a 1.91 Mb segment on RNO7 (Fig 2). Most informative in this regard is the observation that each of the Group 2 congenic strains harbored BN alleles across the 1.91 Mb *Ept7* locus and rats from each of the Group 2 strains exhibited a diminished pituitary growth response to E2 when compared to ACI rats. By contrast, none of the Group 1 congenic strains harbored BN alleles across the 1.91 Mb *Ept7* locus and rats from each Group 1 strain exhibited a pituitary growth response that did not differ significantly from that exhibited by ACI rats. The proximal boundary of this refined *Ept7* locus is defined by microsatellite marker *D7Uwm37* (101.94 Mb on RNO7), at which ACI alleles were observed in the Ept7.18 congenic strain. This proximal boundary is further supported by marker *D7Uwm43* (101.93 Mb on RNO7) which marks the distal end of the BN interval in congenic strains Ept7.9 and Ept7.12 (Fig 2). The distal boundary of the *Ept7* locus is defined by microsatellite marker *D7Rat17*, at which ACI alleles were observed in the Ept7.18 congenic strain as well in several of the other Group 2 congenic strains.

**Fig 3:**
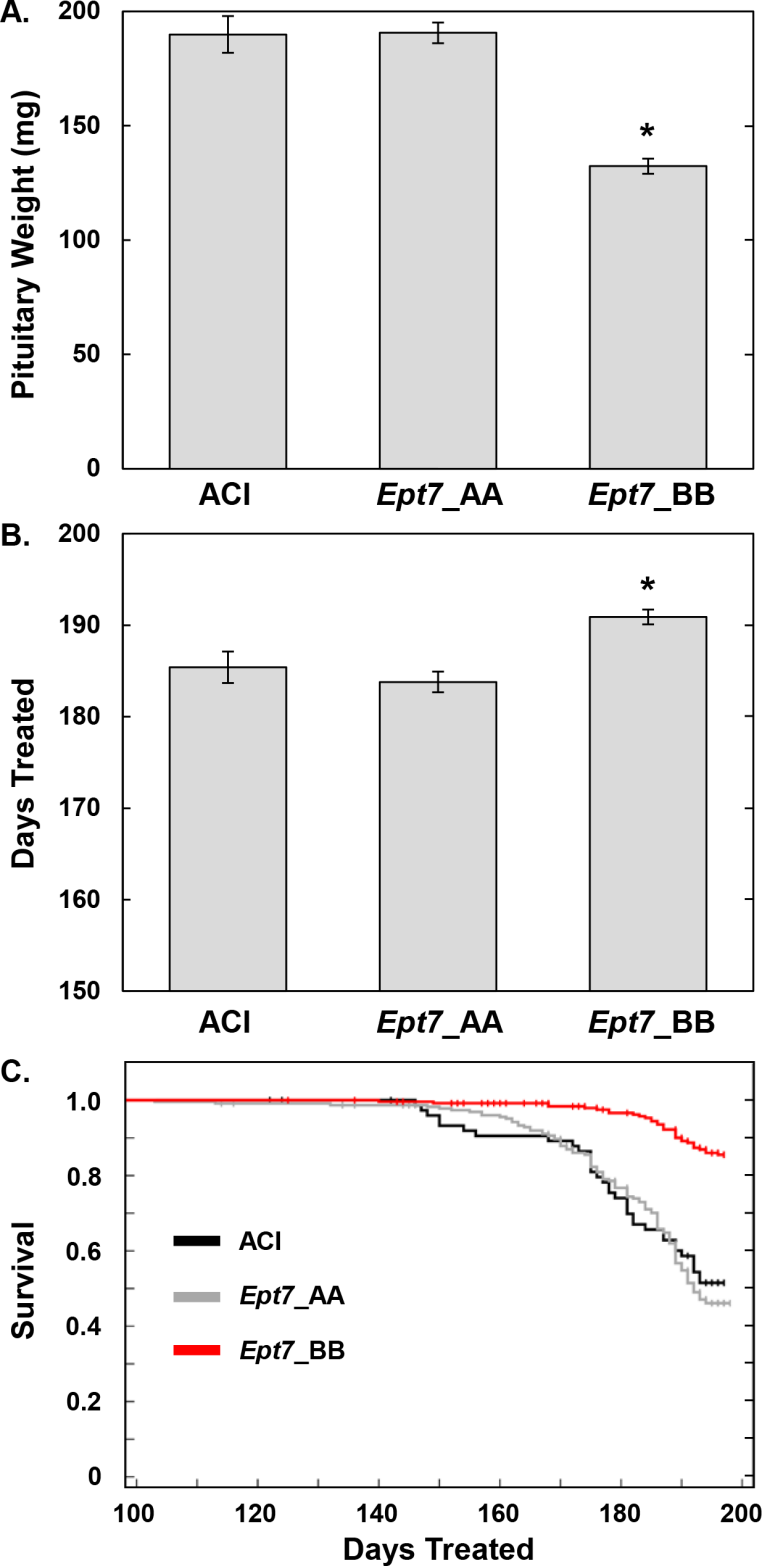
Impact of genotype at *Ept7* on estradiol-induced pituitary growth. Rats from the different congenic strains were pooled based on genotype across the minimal *Ept7* interval. Average pituitary weight (mg ± SEM) (A); average duration of E2 treatment (days ± SEM) (B); and survival over the course of E2 treatment (C) are illustrated for ACI rats (N = 70 (A), 70 (B) and 77 (C)), congenic rats that are homozygous for ACI alleles at *Ept7* (N = 212 (A), 212 (B) and 234 (C)), and congenic rats that are homozygous for BN alleles at *Ept7* (N = 239 (A), 239 (B) and 257 (C)). Animals removed from the study due to mammary cancer related phenotypes were censored in order to clearly illustrate the impact of pituitary tumor related morbidity on survival (ACI, 4 animals censored due to mammary tumor burden; AA congenics, 22 animals censored due to tumor burden; BB congenics, 30 animals censored due to tumor burden).

The Ept7.11 and Ept7.14 congenic strains appear to represent exceptions to the observed linkage between BN alleles across the 1.91 Mb Ept7 locus and reduced pituitary weight. Although Ept7.11 and Ept7.14 rats harbor BN alleles at *Ept7* and exhibited lower average pituitary weights, relative to ACI rats, the observed differences between these congenic rats and ACI rats did not achieve the stringent threshold of statistical significance (P < 0.0025) used to compensate for multiple comparisons (Table 1). However, it is important to note that the Ept7.11 and Ept7.14 rats were treated with E2 for an average of 194.4 and 194.6 days, respectively, which is 9 days longer than the average length of treatment for ACI rats. Because pituitary weight in E2 treated ACI and ACI-derived congenic rats increases as a function of the duration of treatment, we suggest that the 9 day longer duration of E2 treatment partially eclipsed the impact of BN alleles at *Ept7* on induction of pituitary lactotroph hyperplasia and associated pituitary growth during evaluation of the Ept7.11 and Ept7.14 congenic strains. Comparison of congenic rats to ACI rats based solely on genotype at *Ept7* revealed that average pituitary weight in congenic rats harboring BN alleles at *Ept7* was reduced by 57.6 mg (i.e., 30.3%; P = 9.5E-11), relative to ACI rats (Fig 3A). Moreover, the congenic rats harboring BN alleles at *Ept7* were treated with E2 for an average of 5.5 days longer than ACI rats (P = 3.47E-10) (Fig 3B), in large part because of a lower incidence of mortality associated with pituitary enlargement (P = 1.12E-11) (Fig 3C). By contrast, congenic rats harboring ACI alleles at *Ept7* did not differ from ACI rats with respect to pituitary weight (P = 0.99), duration of E2 treatment (P = 0.92), or survival (P = 0.59).

### Genomic features of the *Ept7* locus

**Fig 4:**
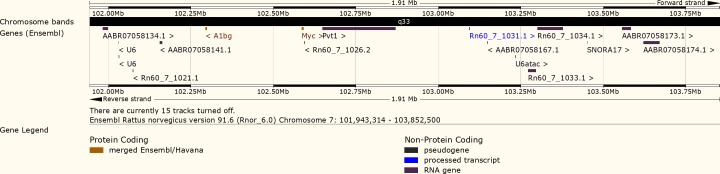
Genome landscape of the *Ept7* locus. This illustration represents the genome annotations assigned within 1.91 Mb *Ept7* locus (figure generated on February 28, 2018 using the Ensembl genome browser and rat genome assembly Rnor_6.0). The annotated features include: 1) two protein coding genes, *A1bg* and *Myc*; 2) *Pvt1*, which yields an assortment of long non-protein coding RNAs; 3) eight predicted genes that may yield long non-coding RNAs (Rn60_7_1031.1, Rn60_7_1033.1, Rn60_7_1034.1, AABR07058134.1, AABR07058141.1, AABR07058167.1, AABR07058173.1, and AABR07058174.1); 4) three predicted genes that may yield small nuclear RNAS (U6, U6, and U6atac); 5) one predicted gene that may yield a small nucleolar RNA (SNORA17); and 6) two processed pseudogenes (Rn60_7_1021.1 and Rn60_7_1026.2).

**Fig 5:**
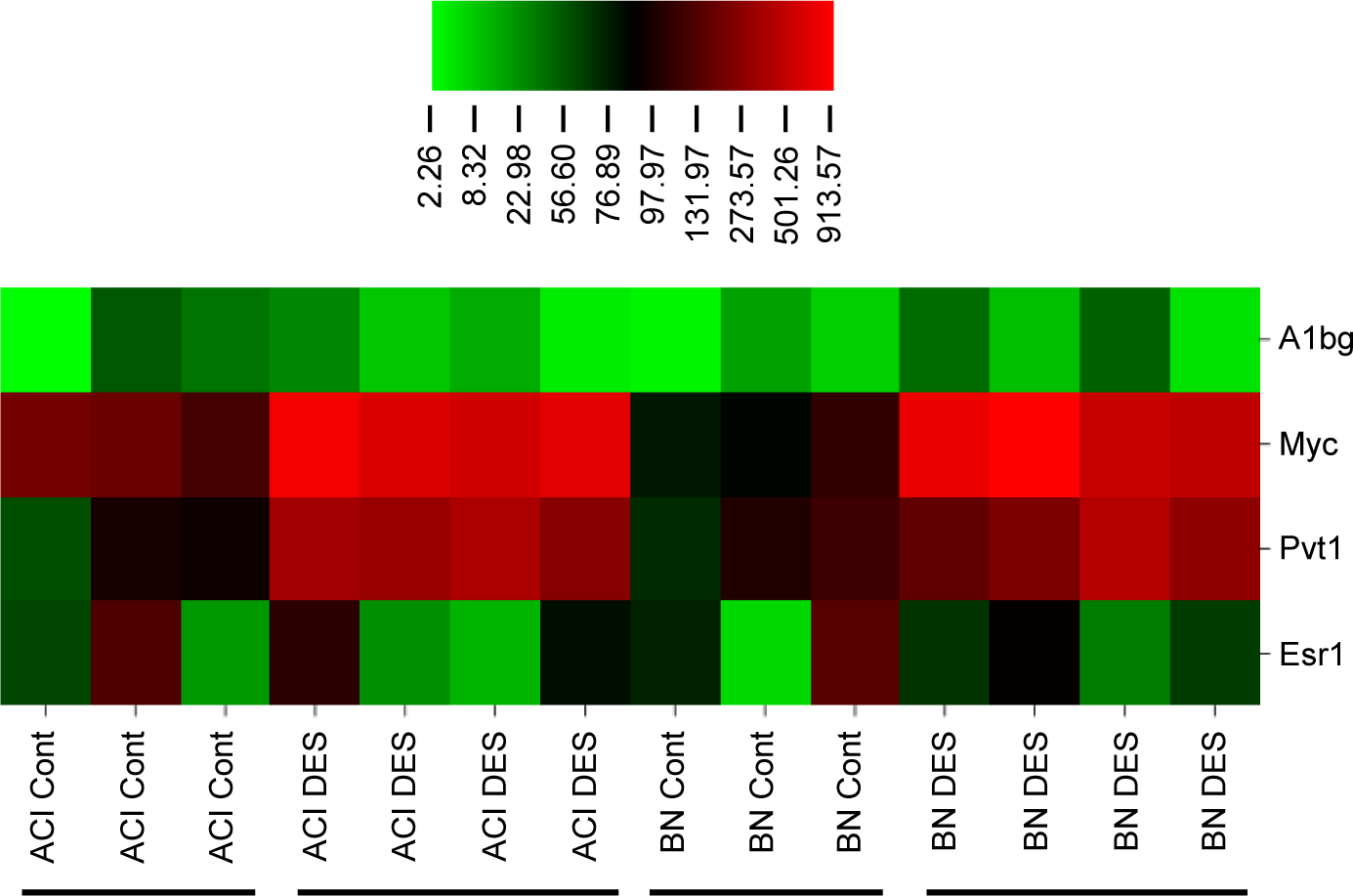
Expression of *Ept7* resident genes. Male ACI and BN rats were treated with the synthetic estrogen diethylstilbestrol (DES), released from subcutaneous Silastic implants, beginning at 9 weeks of age as described previously [33, 36]. Gene expression data were generated from individual rat anterior pituitary glands using Affymetrix GeneChip Rat Genome 230 2.0 Arrays as described previously [40] and the resulting data were deposited in the Gene Expression Omnibus (GEO) database (GEO accession number GDS2913). Data for *A1bg* (probe id = 1369509_a_at), *Myc* (probe id = 1368308_at), and *Pvt1* expression (probe id = 1384449_at) were extracted from this dataset and were illustrated using CIMminer (Genomics and Pharmacology Facility, Developmental Therapeutics Branch, National Cancer Institute, National Institutes of Health). DES treatment significantly increased expression of *Myc* and *Pvt1* in male ACI and BN rats. By contrast, expression of *A1bg* did not differ as a consequence of treatment. Also illustrated are expression data for *Esr1*, which encodes estrogen receptor alpha.

The 1.91 Mb *Ept7* interval harbors two protein coding genes, *A1bg* and *Myc* (Fig 4). *A1bg* encodes alpha-1-B glycoprotein. Searches of the PubMed and GEO databases did not reveal any associations between *A1bg* and estrogen action in the pituitary gland or any other cell or tissue type. Evaluation of gene expression data from our lab (GEO accession number GDS2913) indicates that *A1bg* (probe id = 1369509_a_at) is expressed at a low level in the anterior pituitary gland with no significant differences being apparent between male ACI and BN rats, either sham treated or treated for 12 weeks with the synthetic estrogen diethylstilbestrol (Fig 5). *Myc* encodes Myc proto-oncogene, bHLH transcription factor, which has been functionally linked to development of multiple cancer types. Expression of *Myc* in the anterior pituitary gland is known to be enhanced by estrogens, and *MYC* is highly expressed in prolactinoma relative to normal pituitary [47–49]. Twelve weeks of treatment with diethylstilbestrol increased *Myc* mRNA expression (probe id = 1368308_at) in the anterior pituitary glands of male ACI and BN rats 5-fold (P = 3.8E-4) and 7-fold (P = 6.8E-3), respectively, and no significant differences were observed between the two rat strains. (Fig 5).

The *Ept7* locus also harbors several genes that drive expression of non-protein coding RNAs. Most noteworthy is *Pvt1*, which generates a diverse assortment of alternatively spliced RNAs as well as multiple microRNAs: *miR1204*, *miR1205*, *miR1206*, *miR1207-5p*, and *miR1207-3p*. Microarray data indicate that 12 weeks of treatment with DES increased *Pvt1* expression (probe id = 1384449_at) in the anterior pituitary glands of ACI and BN rats 3.6-fold (P = 9.0E-5) and 2.8-fold (P = 0.017), respectively (Fig 5). *Ept7* also harbors *miR1208*, which is generated from an as yet unidentified primary transcript. Although the miRNAs residing within *Ept7* have not been annotated onto the rat genome, their existence in the rat was predicted and BLAST analyses indicate their presence in the rat in the same relative locations and orientations as in the human and mouse genomes [50]. Finally, it is noted that *D7Rat17*, the marker that defines the distal boundary of *Ept7*, is annotated to two distinct locations on RNO7 separated by 58,383 nucleotides. The duplicated placement of this marker on RNO7 does not impact the results or interpretation of this study.

## Discussion

*Ept7* is one of 19 QTL that have been linked to estrogen-induced increases in pituitary weight, a well-defined surrogate phenotype for lactotroph hyperplasia/adenoma [28, 30, 33, 34, 51]. Data presented in this manuscript refine the location of *Ept7* to a 1.91 Mb interval on RNO7. *Ept7* is the first QTL linked to estrogen action in any cell type, tissue or organ system to be localized to a resolution of less than 2 Mb. The data presented strongly suggest that a genetic variant(s) that resides within *Ept7* impacts expression or function of one or more linked genes and thereby influences estrogen action on the pituitary lactotroph. The actions of the *Ept7* variant(s) could occur directly within the lactotroph itself (i.e., cell autonomous actions). This assertion is supported by *in vivo* and *in vitro* studies that demonstrate that estrogens act directly on the lactotroph to regulate proliferation and prolactin gene expression [25, 52–54]. Expression of at least two of the genes that reside within the *Ept7* locus, *Myc* and *Pvt1*, is increased by administered estrogen *in vivo*. Therefore, we consider *Myc* and *Pvt1* to be strong *Ept7* candidate genes. Alternatively, *Ept7* could act within another estrogen responsive pituitary or extrapituitary cell type that in turn influences lactotroph proliferation; e.g., the tuberoinfundibular neurons of the hypothalamus, which produce and release dopamine, a major negative regulator of lactotroph proliferation and prolactin gene transcription [55–57].

Lactotroph homeostasis is controlled by multiple endocrine factors in addition to estrogens. Prolactin, the primary protein product of the lactotroph, exhibits a wide variety of biological functions, including regulation of mammary gland development and lactation, regulation of maternal behavior, and regulation of the immune system [58]. We postulate that the genetic variants that reside within *Ept7* and the other QTL that control responsiveness of the lactotroph to estrogen exist within the *Rattus norvegicus* population to ensure that a subset of the rat population is able to maintain lactotroph homeostasis under geographical and/or temporal variations in environmental conditions. The pathogenic potential of these genetic variants, as revealed in inbred laboratory rat strains under conditions of prolonged stimulation of estrogen receptor dependent pathways, is informative regarding the molecular mechanisms of estrogen action on the pituitary lactotroph. Because *Homo sapiens* and *Rattus norvegicus* have co-existed and shared environmental challenges over many thousands of years, it is possible that population level variation within orthologous genomic elements in humans and rats may enhance genetic fitness of both species.

Rat models have been employed to map genetic variants that control estrogen action in multiple tissues, including the anterior pituitary [28, 30, 32–36, 59], mammary gland [39, 40, 42, 60], uterus [61, 62], thymus [63], and testis [64]. For the most part, these QTLs map to distinct regions of the rat genome, suggesting the underlying quantitative trait variants influence estrogen action in a cell type specific manner. *Ept7* appears to be the exception in that it localizes to the same region of RNO7 as *Emca4*, a QTL that influences development of estrogen-induced mammary cancer [34, 36, 42]. Data presented localize the *Ept7* quantitative trait variant(s) to the 1.91 Mb interval defined by markers *D7Uwm37* and *D7Rat17*. By contrast, data to be published elsewhere indicate that *Emca4* is a composite QTL that harbors multiple genetic variants that interact with one another to influence development of E2-induced mammary cancers. Although one or more of the *Emca4* quantitative trait variants resides within the same genomic interval as *Ept7*, the others reside proximal to *Ept7* on RNO7. Based upon these observations, it is possible that *Ept7* and *Emca4* may exhibit some overlap in their biological functions.

The *Ept7* locus harbors two annotated protein coding genes, *A1bg* and *Myc*, in addition to genes that generate non-protein coding transcripts, most notably *Pvt1*. Zero published studies were retrieved when the PubMed database was queried using the search terms ‘A1bg and pituitary’ or ‘A1bg and estrogen’. Moreover, although *A1bg* resides proximal to *Myc* in the rat and mouse genomes, in the human genome *A1BG* is not linked to *MYC* on chromosome 8q24 but instead resides on chromosome 19. Consequently, we do not consider *A1bg* to be a high priority *Ept7* candidate.

By contrast, *MYC* is a well characterized protooncogene that is aberrantly overexpressed in many cancer types [65]. Estrogens enhance *Myc* expression in the rat anterior pituitary gland as well as in GH3 rat pituitary tumor cells [49, 66]. Moreover, *MYC* is overexpressed in prolactinomas, relative to the normal anterior pituitary gland [49]. Together, these data suggest that *MYC* may contribute to prolactinoma development in humans and estrogen treated rats. *Pvt1* is representative of a type 3 supergene because it generates a variety of transcripts through use of multiple promoters and alternative transcript splicing/processing to yield multiple functional RNA products [67]. Emerging data suggest the RNA products generated from *Pvt1* coordinately regulate large networks of genes [68]. Multiple studies reveal functional interactions between *MYC* and *PVT1*. For example, *MYC* and *PVT1* are almost always co-amplified in cancers that exhibit somatic copy number gains at 8q24, and mouse models have been used to demonstrate that a single copy gain of a chromosome segment harboring *Myc* and *Pvt1* enhances mammary tumorigenesis driven by an *MMTV-NEU* transgene, whereas single copy gains of *Myc* or *Pvt1* alone do not [69]. In addition, MYC has been demonstrated to activate transcription from the *PVT1* promoter in cultured cells [70]. More recently, the *MYC* and *PVT1* promoters have been shown to compete for engagement with a set of enhancers located within *PVT1* [71]. We are working to define the functions of *Pvt1* and the different miRNAs residing within the *Ept7* locus in the rat mammary and pituitary glands.

Data presented herein indicate the *Ept7* locus harbors a variant(s) that influences the actions of estrogens in the regulation of pituitary lactotroph proliferation as reflected in development of E2-induced lactotroph hyperplasia/adenoma. Data to be published elsewhere indicate that a variant(s) within the same genome interval are part of a multipartite genetic element that controls estrogen action in the rat mammary gland and influences susceptibility to E2-induced mammary cancer. The region of the human genome that is orthologous to *Ept7* has been linked in genome wide association studies to multiple cancer types, developmental defects, and physiological phenotypes (S2 Table). For example, SNPs residing within the *Ept7* orthologous region have been associated with breast cancer [72–74], endometrial cancer [75], and epithelial and high grade serous ovarian cancers [76]. Estrogens have been implicated in the development of each of these cancer types. Therefore, molecular characterization of the genes and variants that reside in *Ept7* may yield novel insights into the etiology of multiple diseases.

## Supporting information

S1 Fig Pituitary weights in E2 treated ACI females by date of necropsy. Fifteen groups of ACI females were evaluated starting at different points over a five-year period contemporaneous to their congenic counterparts. Female rats were treated with E2, released from subcutaneous Silastic implants, beginning at 9 weeks of age as described in Materials and Methods. Animals were euthanized upon observation of any treatment associated morbidity or following 28 weeks of treatment. Pituitary weight measured at necropsy is indicated on the y-axis; date of necropsy is indicated on the x-axis (month-year). Each data point represents one individual. The dashed line indicates the mean for the population (189.9 mg); the shaded region demarcates one standard deviation from the mean (± 67.0 mg). The dotted line indicates best fit by linear regression in Microsoft Excel 2016 (y = -0.0157x + 832.19); N = 70.
(TIF)

S1 Table Genetic characteristics of Ept7 congenic strains. (DOCX)

S2 Table SNPs within human *Ept7* orthologous region associated (GWAS) with disease and normal physiologic phenotypes. (XLSX)

## References

[1] Molitch ME. Diagnosis and Treatment of Pituitary Adenomas: A Review. JAMA: the journal of the American Medical Association. 2017;317(5):516–24. 10.1001/jama.2016.19699 . 28170483

[2] Melmed S. Pathogenesis of pituitary tumors. Nature reviews Endocrinology. 2011;7(5):257–66. 10.1038/nrendo.2011.40 . 21423242

[3] Asa SL, Ezzat S. The pathogenesis of pituitary tumors. Annu Rev Pathol. 2009;4:97–126. 10.1146/annurev.pathol.4.110807.092259 . 19400692

[4] Dworakowska D, Grossman AB. The pathophysiology of pituitary adenomas. Best practice & research Clinical endocrinology & metabolism. 2009;23(5):525–41. 10.1016/j.beem.2009.05.004 . 19945021

[5] Chahal HS, Chapple JP, Frohman LA, Grossman AB, Korbonits M. Clinical, genetic and molecular characterization of patients with familial isolated pituitary adenomas (FIPA). Trends in endocrinology and metabolism: TEM. 2010;21(7):419–27. 10.1016/j.tem.2010.02.007 . 20570174

[6] Molitch ME. Pituitary disorders during pregnancy. Endocrinology and metabolism clinics of North America. 2006;35(1):99–116, vi. 10.1016/j.ecl.2005.09.011 . 16310644

[7] Casanueva FF, Molitch ME, Schlechte JA, Abs R, Bonert V, Bronstein MD, et al Guidelines of the Pituitary Society for the diagnosis and management of prolactinomas. Clinical endocrinology. 2006;65(2):265–73. 10.1111/j.1365-2265.2006.02562.x . 16886971

[8] Colao A, Savastano S. Medical treatment of prolactinomas. Nature reviews Endocrinology. 2011;7(5):267–78. 10.1038/nrendo.2011.37 . 21423245

[9] Gooren LJ, Assies J, Asscheman H, de Slegte R, van Kessel H. Estrogen-induced prolactinoma in a man. The Journal of clinical endocrinology and metabolism. 1988;66(2):444–6. 10.1210/jcem-66-2-444 . 3339116

[10] Asscheman H, Gooren LJ, Assies J, Smits JP, de Slegte R. Prolactin levels and pituitary enlargement in hormone-treated male-to-female transsexuals. Clinical endocrinology. 1988;28(6):583–8. . 2978262 10.1111/j.1365-2265.1988.tb03849.x

[11] Kovacs K, Stefaneanu L, Ezzat S, Smyth HS. Prolactin-producing pituitary adenoma in a male-to-female transsexual patient with protracted estrogen administration. A morphologic study. Archives of pathology & laboratory medicine. 1994;118(5):562–5. . 8192565

[12] Serri O, Noiseux D, Robert F, Hardy J. Lactotroph hyperplasia in an estrogen treated male-to-female transsexual patient. The Journal of clinical endocrinology and metabolism. 1996;81(9):3177–9. 10.1210/jcem.81.9.8784065 . 8784065

[13] Gooren LJ, Giltay EJ, Bunck MC. Long-term treatment of transsexuals with cross-sex hormones: extensive personal experience. The Journal of clinical endocrinology and metabolism. 2008;93(1):19–25. 10.1210/jc.2007-1809 . 17986639

[14] Mueller A, Gooren L. Hormone-related tumors in transsexuals receiving treatment with cross-sex hormones. European journal of endocrinology / European Federation of Endocrine Societies. 2008;159(3):197–202. 10.1530/EJE-08-0289 . 18567667

[15] Bunck MC, Debono M, Giltay EJ, Verheijen AT, Diamant M, Gooren LJ. Autonomous prolactin secretion in two male-to-female transgender patients using conventional oestrogen dosages. BMJ case reports. 2009;2009 10.1136/bcr.02.2009.1589 ; PubMed Central PMCID: PMC3029513. 21829422 PMC3029513

[16] Garcia-Malpartida K, Martin-Gorgojo A, Rocha M, Gomez-Balaguer M, Hernandez-Mijares A. Prolactinoma induced by estrogen and cyproterone acetate in a male-to-female transsexual. Fertility and sterility. 2010;94(3):1097 e13–5. 10.1016/j.fertnstert.2010.01.076 . 20227072

[17] Cunha FS, Domenice S, Camara VL, Sircili MH, Gooren LJ, Mendonca BB, et al Diagnosis of prolactinoma in two male-to-female transsexual subjects following high-dose cross-sex hormone therapy. Andrologia. 2014 10.1111/and.12317 . 25059808

[18] Gorski J, Wendell D, Gregg D, Chun TY. Estrogens and the genetic control of tumor growth. Progress in clinical and biological research. 1997;396:233–43. . 9108601

[19] Sarkar DK, Hentges ST, De A, Reddy RH. Hormonal control of pituitary prolactin-secreting tumors. Frontiers in bioscience: a journal and virtual library. 1998;3:d934–43. . 9696884 10.2741/a334

[20] Spady TJ, McComb RD, Shull JD. Estrogen action in the regulation of cell proliferation, cell survival, and tumorigenesis in the rat anterior pituitary gland. Endocrine. 1999;11(3):217–33. Epub 2000/04/29. 10.1385/ENDO:11:3:217 . 10786818

[21] Sarkar DK. Genesis of prolactinomas: studies using estrogen-treated animals. Frontiers of hormone research. 2006;35:32–49. 10.1159/000094307 ; PubMed Central PMCID: PMC2882189. 16809921 PMC2882189

[22] Stone JP, Holtzman S, Shellabarger CJ. Neoplastic responses and correlated plasma prolactin levels in diethylstilbestrol-treated ACI and Sprague-Dawley rats. Cancer research. 1979;39(3):773–8. Epub 1979/03/01. . 427764

[23] Holtzman S, Stone JP, Shellabarger CJ. Influence of diethylstilbestrol treatment on prolactin cells of female ACI and Sprague-Dawley rats. Cancer research. 1979;39(3):779–84. . 427765

[24] Wiklund J, Rutledge J, Gorski J. A genetic model for the inheritance of pituitary tumor susceptibility in F344 rats. Endocrinology. 1981;109(5):1708–14. 10.1210/endo-109-5-1708 . 7297501

[25] Wiklund J, Wertz N, Gorski J. A comparison of estrogen effects on uterine and pituitary growth and prolactin synthesis in F344 and Holtzman rats. Endocrinology. 1981;109(5):1700–7. 10.1210/endo-109-5-1700 . 7297500

[26] Wiklund JA, Gorski J. Genetic differences in estrogen-induced deoxyribonucleic acid synthesis in the rat pituitary: correlations with pituitary tumor susceptibility. Endocrinology. 1982;111(4):1140–9. 10.1210/endo-111-4-1140 . 7117195

[27] Wendell DL, Herman A, Gorski J. Genetic separation of tumor growth and hemorrhagic phenotypes in an estrogen-induced tumor. Proceedings of the National Academy of Sciences of the United States of America. 1996;93(15):8112–6. ; PubMed Central PMCID: PMC38884. 8755612 10.1073/pnas.93.15.8112 PMC38884

[28] Wendell DL, Gorski J. Quantitative trait loci for estrogen-dependent pituitary tumor growth in the rat. Mammalian genome: official journal of the International Mammalian Genome Society. 1997;8(11):823–9. . 9337394 10.1007/s003359900586

[29] Spady TJ, Pennington KL, McComb RD, Shull JD. Genetic bases of estrogen-induced pituitary growth in an intercross between the ACI and Copenhagen rat strains: dominant mendelian inheritance of the ACI phenotype. Endocrinology. 1999;140(6):2828–35. Epub 1999/05/26. 10.1210/endo.140.6.6757 . 10342874

[30] Wendell DL, Daun SB, Stratton MB, Gorski J. Different functions of QTL for estrogen-dependent tumor growth of the rat pituitary. Mammalian genome: official journal of the International Mammalian Genome Society. 2000;11(10):855–61. . 11003699 10.1007/s003350010168

[31] Sclafani RV, Wendell DL. Suppression of estrogen-dependent MMP-9 expression by Edpm5, a genetic locus for pituitary tumor growth in rat. Molecular and cellular endocrinology. 2001;176(1–2):145–53. . 11369454 10.1016/s0303-7207(01)00401-4

[32] Pandey J, Bannout A, Wendell DL. The Edpm5 locus prevents the 'angiogenic switch' in an estrogen-induced rat pituitary tumor. Carcinogenesis. 2004;25(10):1829–38. 10.1093/carcin/bgh192 . 15166088

[33] Strecker TE, Spady TJ, Schaffer BS, Gould KA, Kaufman AE, Shen F, et al Genetic bases of estrogen-induced pituitary tumorigenesis: identification of genetic loci determining estrogen-induced pituitary growth in reciprocal crosses between the ACI and Copenhagen rat strains. Genetics. 2005;169(4):2189–97. Epub 2005/02/03. 10.1534/genetics.104.039370 ; PubMed Central PMCID: PMC1449615. 15687265 PMC1449615

[34] Shull JD, Lachel CM, Murrin CR, Pennington KL, Schaffer BS, Strecker TE, et al Genetic control of estrogen action in the rat: mapping of QTLs that impact pituitary lactotroph hyperplasia in a BN x ACI intercross. Mammalian genome: official journal of the International Mammalian Genome Society. 2007;18(9):657–69. Epub 2007/09/19. 10.1007/s00335-007-9052-2 . 17876666

[35] Kurz SG, Hansen KK, McLaughlin MT, Shivaswamy V, Schaffer BS, Gould KA, et al Tissue-specific actions of the Ept1, Ept2, Ept6, and Ept9 genetic determinants of responsiveness to estrogens in the female rat. Endocrinology. 2008;149(8):3850–9. Epub 2008/04/19. 10.1210/en.2008-0173 ; PubMed Central PMCID: PMC2488241. 18420736 PMC2488241

[36] Kurz SG, Dennison KL, Samanas NB, Hickman MP, Eckert QA, Walker TL, et al Ept7 influences estrogen action in the pituitary gland and body weight of rats. Mammalian genome: official journal of the International Mammalian Genome Society. 2014;25(5–6):244–52. Epub 2014/01/23. 10.1007/s00335-014-9504-4 ; PubMed Central PMCID: PMC4035442. 24448715 PMC4035442

[37] Guide for the Care and Use of Laboratory Animals. Eighth ed. Washington, D.C.: National Academies Press; 2011. 21595115

[38] Dennison KL, Samanas NB, Harenda QE, Hickman MP, Seiler NL, Ding L, et al Development and characterization of a novel rat model of estrogen-induced mammary cancer. Endocrine-related cancer. 2015;22(2):239–48. 10.1530/ERC-14-0539 ; PubMed Central PMCID: PMC4372900. 25800038 PMC4372900

[39] Colletti JA 2nd, Leland-Wavrin KM, Kurz SG, Hickman MP, Seiler NL, Samanas NB, et al Validation of six genetic determinants of susceptibility to estrogen-induced mammary cancer in the rat and assessment of their relevance to breast cancer risk in humans. G3. 2014;4(8):1385–94. 10.1534/g3.114.011163 ; PubMed Central PMCID: PMC4132170. 24875630 PMC4132170

[40] Schaffer BS, Leland-Wavrin KM, Kurz SG, Colletti JA, Seiler NL, Warren CL, et al Mapping of three genetic determinants of susceptibility to estrogen-induced mammary cancer within the Emca8 locus on rat chromosome 5. Cancer prevention research. 2013;6(1):59–69. Epub 2012/11/16. 10.1158/1940-6207.CAPR-12-0346-T ; PubMed Central PMCID: PMC3536887. 23151807 PMC3536887

[41] Ding L, Zhao Y, Warren CL, Sullivan R, Eliceiri KW, Shull JD. Association of cellular and molecular responses in the rat mammary gland to 17beta-estradiol with susceptibility to mammary cancer. BMC cancer. 2013;13:573 Epub 2013/12/07. 10.1186/1471-2407-13-573 . 24304664 PMC3924185

[42] Schaffer BS, Lachel CM, Pennington KL, Murrin CR, Strecker TE, Tochacek M, et al Genetic bases of estrogen-induced tumorigenesis in the rat: mapping of loci controlling susceptibility to mammary cancer in a Brown Norway x ACI intercross. Cancer research. 2006;66(15):7793–800. Epub 2006/08/04. 10.1158/0008-5472.CAN-06-0143 . 16885383

[43] Drinkwater NR. MSTAT Version 6.4.2 2018. Available from: http://www.mcardle.wisc.edu/mstat/index.html.

[44] Nigam R, Laulederkind SJ, Hayman GT, Smith JR, Wang SJ, Lowry TF, et al Rat Genome Database: a unique resource for rat, human, and mouse quantitative trait locus data. Physiological genomics. 2013;45(18):809–16. Epub 2013/07/25. 10.1152/physiolgenomics.00065.2013 ; PubMed Central PMCID: PMC3783816. 23881287 PMC3783816

[45] Laulederkind SJ, Hayman GT, Wang SJ, Smith JR, Lowry TF, Nigam R, et al The Rat Genome Database 2013—data, tools and users. Briefings in bioinformatics. 2013;14(4):520–6. Epub 2013/02/26. 10.1093/bib/bbt007 ; PubMed Central PMCID: PMC3713714. 23434633 PMC3713714

[46] Zerbino DR, Achuthan P, Akanni W, Amode MR, Barrell D, Bhai J, et al Ensembl 2018. Nucleic acids research. 2018;46(D1):D754–D61. Epub 2017/11/21. 10.1093/nar/gkx1098 ; PubMed Central PMCID: PMCPMC5753206. 29155950 PMC5753206

[47] Szijan I, Parma DL, Engel NI. Expression of c-myc and c-fos protooncogenes in the anterior pituitary gland of the rat. Effect of estrogen. Hormone and metabolic research = Hormon- und Stoffwechselforschung = Hormones et metabolisme. 1992;24(4):154–7. 10.1055/s-2007-1003283 . 1601388

[48] Chernavsky AC, Valerani AV, Burdman JA. Haloperidol and oestrogens induce c-myc and c-fos expression in the anterior pituitary gland of the rat. Neurological research. 1993;15(5):339–43. . 7905609 10.1080/01616412.1993.11740158

[49] Tong Y, Zheng Y, Zhou J, Oyesiku NM, Koeffler HP, Melmed S. Genomic characterization of human and rat prolactinomas. Endocrinology. 2012;153(8):3679–91. 10.1210/en.2012-1056 ; PubMed Central PMCID: PMC3404356. 22635680 PMC3404356

[50] Huppi K, Volfovsky N, Runfola T, Jones TL, Mackiewicz M, Martin SE, et al The identification of microRNAs in a genomically unstable region of human chromosome 8q24. Molecular cancer research: MCR. 2008;6(2):212–21. 10.1158/1541-7786.MCR-07-0105 . 18314482

[51] Jerry DJ, Shull JD, Hadsell DL, Rijnkels M, Dunphy KA, Schneider SS, et al Genetic variation in sensitivity to estrogens and breast cancer risk. Mammalian genome: official journal of the International Mammalian Genome Society. 2018;29(1–2):24–37. Epub 2018/03/01. 10.1007/s00335-018-9741-z . 29487996 PMC5936622

[52] Lieberman ME, Maurer RA, Claude P, Wiklund J, Wertz N, Gorski J. Regulation of pituitary growth and prolactin gene expression by estrogen. Advances in experimental medicine and biology. 1981;138:151–63. . 7342713 10.1007/978-1-4615-7192-6_9

[53] Shull JD, Gorski J. The hormonal regulation of prolactin gene expression: an examination of mechanisms controlling prolactin synthesis and the possible relationship of estrogen to these mechanisms. Vitamins and hormones. 1986;43:197–249. Epub 1986/01/01. . 2431543 10.1016/s0083-6729(08)60421-5

[54] Shull JD, Walent JH, Gorski J. Estradiol stimulates prolactin gene transcription in primary cultures of rat anterior pituitary cells. Journal of steroid biochemistry. 1987;26(4):451–6. Epub 1987/04/01. . 3586660 10.1016/0022-4731(87)90055-0

[55] Spady TJ, Harvell DM, Lemus-Wilson A, Strecker TE, Pennington KL, Vander Woude EA, et al Modulation of estrogen action in the rat pituitary and mammary glands by dietary energy consumption. The Journal of nutrition. 1999;129(2S Suppl):587S–90S. Epub 1999/03/04. 10.1093/jn/129.2.587S . 10064338

[56] Shaw-Bruha CM, Happe HK, Murrin LC, Rodriguez-Sierra JF, Shull JD. 17 beta-Estradiol inhibits the production of dopamine by the tuberoinfundibular dopaminergic neurons of the male rat. Brain research bulletin. 1996;40(1):33–6. Epub 1996/01/01. . 8722750 10.1016/0361-9230(96)00004-4

[57] Shull JD, Gorski J. Regulation of prolactin gene transcription in vivo: interactions between estrogen, pimozide, and alpha-ergocryptine. Molecular pharmacology. 1990;37(2):215–21. Epub 1990/02/01. . 1968222

[58] Goffin V, Binart N, Touraine P, Kelly PA. Prolactin: the new biology of an old hormone. Annu Rev Physiol. 2002;64:47–67. Epub 2002/02/05. 10.1146/annurev.physiol.64.081501.131049 . 11826263

[59] Wendell DL, Pandey J, Kelley P. A congenic strain of rat for investigation of control of estrogen-induced growth. Mammalian genome: official journal of the International Mammalian Genome Society. 2002;13(11):664–6. 10.1007/s00335-002-2183-6 . 12461653

[60] Gould KA, Tochacek M, Schaffer BS, Reindl TM, Murrin CR, Lachel CM, et al Genetic determination of susceptibility to estrogen-induced mammary cancer in the ACI rat: mapping of Emca1 and Emca2 to chromosomes 5 and 18. Genetics. 2004;168(4):2113–25. Epub 2004/12/22. 10.1534/genetics.104.033878 ; PubMed Central PMCID: PMC1448731. 15611180 PMC1448731

[61] Gould KA, Pandey J, Lachel CM, Murrin CR, Flood LA, Pennington KL, et al Genetic mapping of Eutr1, a locus controlling E2-induced pyometritis in the Brown Norway rat, to RNO5. Mammalian genome: official journal of the International Mammalian Genome Society. 2005;16(11):854–64. Epub 2005/11/15. 10.1007/s00335-005-0070-7 . 16284801

[62] Pandey J, Gould KA, McComb RD, Shull JD, Wendell DL. Localization of Eutr2, a locus controlling susceptibility to DES-induced uterine inflammation and pyometritis, to RNO5 using a congenic rat strain. Mammalian genome: official journal of the International Mammalian Genome Society. 2005;16(11):865–72. Epub 2005/11/15. 10.1007/s00335-005-0071-6 . 16284802

[63] Gould KA, Strecker TE, Hansen KK, Bynote KK, Peterson KA, Shull JD. Genetic mapping of loci controlling diethylstilbestrol-induced thymic atrophy in the Brown Norway rat. Mammalian genome: official journal of the International Mammalian Genome Society. 2006;17(5):451–64. Epub 2006/05/12. 10.1007/s00335-005-0183-z . 16688534

[64] Tachibana M, Lu L, Hiai H, Tamura A, Matsushima Y, Shisa H. Quantitative trait loci determining weight reduction of testes and pituitary by diethylstilbesterol in LEXF and FXLE recombinant inbred strain rats. Experimental animals / Japanese Association for Laboratory Animal Science. 2006;55(2):91–5. . 16651691 10.1538/expanim.55.91

[65] Carroll PA, Freie BW, Mathsyaraja H, Eisenman RN. The MYC transcription factor network: balancing metabolism, proliferation and oncogenesis. Front Med. 2018 Epub 2018/07/29. 10.1007/s11684-018-0650-z . 30054853 PMC7358075

[66] Fujimoto N, Igarashi K, Kanno J, Inoue T. Identification of estrogen-responsive genes in the GH3 cell line by cDNA microarray analysis. The Journal of steroid biochemistry and molecular biology. 2004;91(3):121–9. Epub 2004/07/28. 10.1016/j.jsbmb.2004.02.006 . 15276619

[67] Anastasiadou E, Jacob LS, Slack FJ. Non-coding RNA networks in cancer. Nature reviews Cancer. 2018;18(1):5–18. 10.1038/nrc.2017.99 . 29170536 PMC6337726

[68] Paci P, Colombo T, Farina L. Computational analysis identifies a sponge interaction network between long non-coding RNAs and messenger RNAs in human breast cancer. BMC systems biology. 2014;8:83 10.1186/1752-0509-8-83 ; PubMed Central PMCID: PMC4113672. 25033876 PMC4113672

[69] Tseng YY, Moriarity BS, Gong W, Akiyama R, Tiwari A, Kawakami H, et al PVT1 dependence in cancer with MYC copy-number increase. Nature. 2014;512(7512):82–6. 10.1038/nature13311 . 25043044 PMC4767149

[70] Carramusa L, Contino F, Ferro A, Minafra L, Perconti G, Giallongo A, et al The PVT-1 oncogene is a Myc protein target that is overexpressed in transformed cells. Journal of cellular physiology. 2007;213(2):511–8. 10.1002/jcp.21133 . 17503467

[71] Cho SW, Xu J, Sun R, Mumbach MR, Carter AC, Chen YG, et al Promoter of lncRNA Gene PVT1 Is a Tumor-Suppressor DNA Boundary Element. Cell. 2018;173(6):1398–412 e22. Epub 2018/05/08. 10.1016/j.cell.2018.03.068 ; PubMed Central PMCID: PMCPMC5984165. 29731168 PMC5984165

[72] Michailidou K, Beesley J, Lindstrom S, Canisius S, Dennis J, Lush MJ, et al Genome-wide association analysis of more than 120,000 individuals identifies 15 new susceptibility loci for breast cancer. Nature genetics. 2015;47(4):373–80. 10.1038/ng.3242 . 25751625 PMC4549775

[73] Michailidou K, Hall P, Gonzalez-Neira A, Ghoussaini M, Dennis J, Milne RL, et al Large-scale genotyping identifies 41 new loci associated with breast cancer risk. Nature genetics. 2013;45(4):353–61, 61e1-2. Epub 2013/03/29. 10.1038/ng.2563 ; PubMed Central PMCID: PMC3771688. 23535729 PMC3771688

[74] Michailidou K, Lindstrom S, Dennis J, Beesley J, Hui S, Kar S, et al Association analysis identifies 65 new breast cancer risk loci. Nature. 2017;551(7678):92–4. 10.1038/nature24284 . 29059683 PMC5798588

[75] Cheng TH, Thompson DJ, O'Mara TA, Painter JN, Glubb DM, Flach S, et al Five endometrial cancer risk loci identified through genome-wide association analysis. Nature genetics. 2016;48(6):667–74. 10.1038/ng.3562 ; PubMed Central PMCID: PMCPMC4907351. 27135401 PMC4907351

[76] Phelan CM, Kuchenbaecker KB, Tyrer JP, Kar SP, Lawrenson K, Winham SJ, et al Identification of 12 new susceptibility loci for different histotypes of epithelial ovarian cancer. Nature genetics. 2017;49(5):680–91. 10.1038/ng.3826 ; PubMed Central PMCID: PMCPMC5612337. 28346442 PMC5612337

