# Supplementary figures and images for: Ept7, a quantitative trait locus that controls estrogen-induced pituitary lactotroph hyperplasia in rat, is orthologous to a locus in humans that has been associated with numerous cancer types and common diseases
Source: PLoS One. 2018 Sep 27;13(9):e0204727. doi: 10.1371/journal.pone.0204727 (PMC6160183; doi:10.1371/journal.pone.0204727)

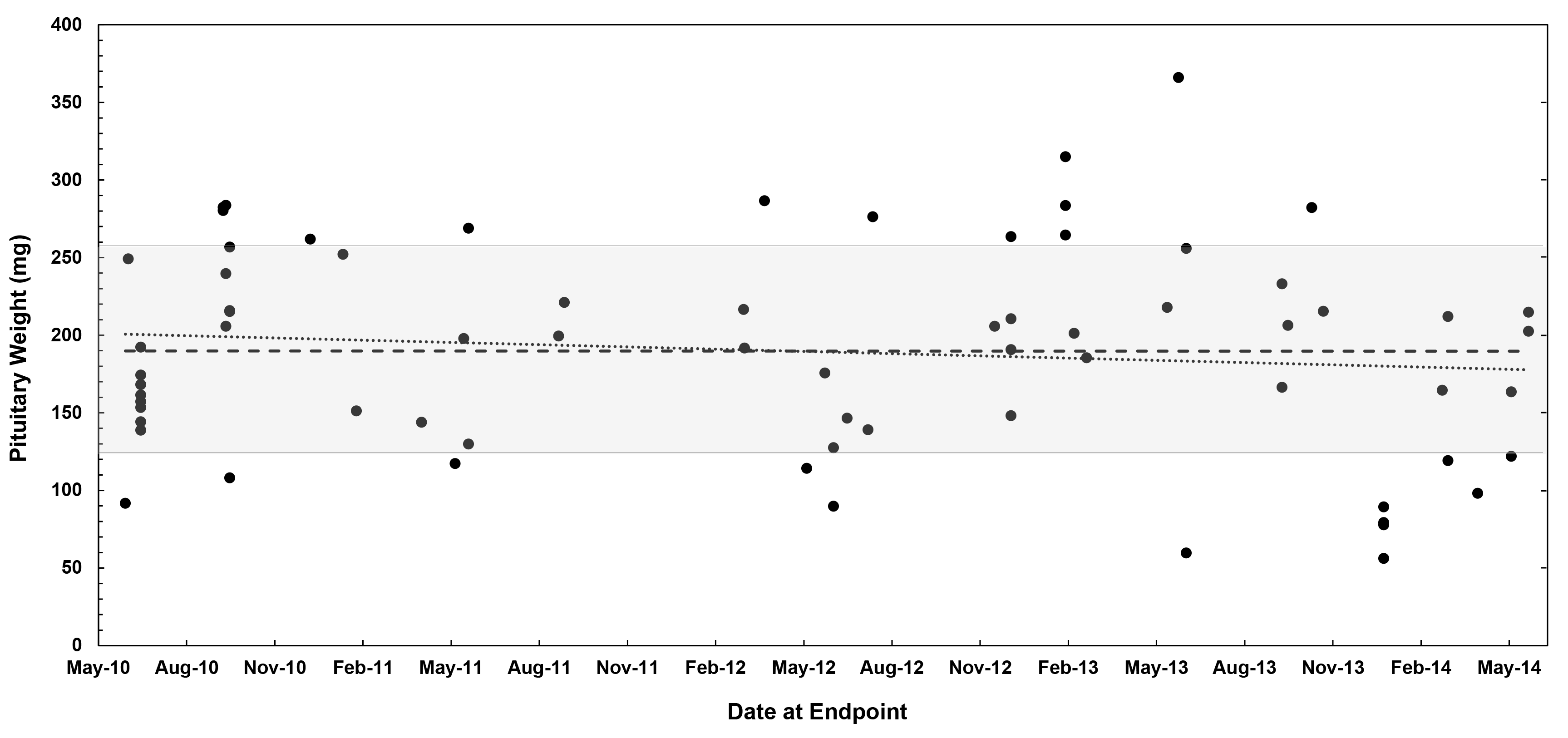

Supplement: S1 Fig — Fifteen groups of ACI females were evaluated starting at different points over a five-year period contemporaneous to their congenic counterparts. Female rats were treated with E2, released from subcutaneous Silastic implants, beginning at 9 weeks of age as described in Materials and Methods. Animals were euthanized upon observation of any treatment associated morbidity or following 28 weeks of treatment. Pituitary weight measured at necropsy is indicated on the y-axis; date of necropsy is indicated on the x-axis (month-year). Each data point represents one individual. The dashed line indicates the mean for the population (189.9 mg); the shaded region demarcates one standard deviation from the mean (± 67.0 mg). The dotted line indicates best fit by linear regression in Microsoft Excel 2016 (y = -0.0157x + 832.19); N = 70. (TIF) [file pone.0204727.s001.tif]
