# Supplementary material for: Ept7, a quantitative trait locus that controls estrogen-induced pituitary lactotroph hyperplasia in rat, is orthologous to a locus in humans that has been associated with numerous cancer types and common diseases
Source: PLoS One. 2018 Sep 27;13(9):e0204727. doi: 10.1371/journal.pone.0204727 (PMC6160183; doi:10.1371/journal.pone.0204727)
Supplement: S1 Table — (DOCX) [file pone.0204727.s002.docx]

**S1 Table. Genetic Characteristics of Ept7 Congenic Strains**

| **Strain Designation** | **Strain Abbreviation** | **Proximal**  **ACI** | **Proximal**  **BN** | **Distal**  **BN** | **Distal**  **ACI** | **RGD ID^1^** |
| --- | --- | --- | --- | --- | --- | --- |
| ACI.BN-(*D7Rat164-D7Uwm27*)/Shul | Ept7 | *D7Wox3*  71.45 Mb | *D7Rat164*  72.75 Mb | *D7Uwm27*  103.64 Mb | *D7Rat17^2^*  103.85 Mb | 7248725 |
| ACI.BN-(D7Rat164-D7Uwm33)/Shul | Ept7.1 | *D7Wox3*  71.45 Mb | *D7Rat164*  72.75 Mb | *D7Uwm33*  97.35 Mb | *D7Arb15*  100.16 Mb | 7248733 |
| ACI.BN-(D7Uwm41-D7Mit16)/Shul | Ept7.2 | *D7Arb15*  100.16 Mb | *D7Uwm41*  100.59 Mb | *D7Mit16*  123.60 Mb | *D7Rat9*  128.09 Mb | 7248746 |
| ACI.BN-(D7Uwm28-D7Mit16)/Shul | Ept7.3 | *D7Uwm41*  100.59 Mb | *D7Uwm28*  101.68 Mb | *D7Mit16*  123.60 Mb | *D7Rat9*  128.09 Mb | 7248748 |
| ACI.BN-(*D7Rat164-D7Uwm30*)/Shul | Ept7.4 | *D7Wox3*  71.45 Mb | *D7Rat164*  72.75 Mb | *D7Uwm30*  83.37 Mb | *D7Got50*  85.27 Mb | 7248727 |
| ACI.BN-(D7Rat42-D7Uwm33)/Shul | Ept7.5 | *D7Mit5*  88.37 Mb | *D7Rat42*  92.23 Mb | *D7Uwm33*  97.35 Mb | *rgdv775930055-A^3^*  99.73 Mb | 7364932 |
| ACI.BN-(*D7Uwm32*-*D7Uwm27*)/Shul | Ept7.6 | *D7Uwm34*  94.38 Mb | *D7Uwm32*  94.64 Mb | *D7Uwm27*  103.64 Mb | *D7Rat17^2^*  103.85 Mb | 7248736 |
| ACI.BN-(*D7Rat164-D7Rat142*)/Shul | Ept7.7 | *D7Wox3*  71.45 Mb | *D7Rat164*  72.75 Mb | *D7Rat142*  79.63 Mb | *D7Uwm42*  80.76 Mb | 8663458 |
| ACI.BN-(*D7Rat206-D7Uwm30*)/Shul | Ept7.8 | *rgdv877215464-A^3^*  77.37 | *D7Rat206*  78.76 | *D7Uwm30*  83.37 Mb | *D7Got50*  85.27 Mb | 7248729 |
| ACI.BN-(*D7Uwm32-D7Uwm43*)/Shul | Ept7.9 | *D7Uwm34*  94.38 Mb | *D7Uwm32*  94.64 Mb | *D7Uwm43*  101.93 Mb | *D7Uwm37*  101.94 Mb | 8663462 |
| ACI.BN-(D7Uwm33-D7Uwm27)/Shul | Ept7.10 | *D7Uwm39*  96.47 Mb | *D7Uwm33*  97.35 Mb | *D7Uwm27*  103.64 Mb | *D7Rat17^2^*  103.85 Mb | 7248744 |
| ACI.BN-(*249431173-D7Uwm27*)/Shul | Ept7.11 | *rgdv775925571-T^3^*  97.46 Mb | *rgdv775925951-C^3^*  98.51 Mb | *D7Uwm27*  103.64 Mb | *D7Rat17^2^*  103.85 Mb | 13210773 |
| ACI.BN-(*D7Uwm33-D7Uwm43*)/Shul | Ept7.12 | *D7Uwm39*  96.47 Mb | *D7Uwm33*  97.35 Mb | *D7Uwm43*  101.93 Mb | *D7Uwm37*  101.94 Mb | 8663464 |
| ACI.BN-(*D7Rat164-D7Uwm31*)/Shul | Ept7.13 | *D7Wox3*  71.45 Mb | *D7Rat164*  72.75 Mb | *D7Uwm31*  76.14 Mb | *rs198654169*  76.86 Mb | 7248731 |
| ACI.BN-(D7Arb15-D7Uwm27)/Shul | Ept7.14 | *rgdv775925951-C^3^*  98.51 Mb | *D7Arb15*  100.16 Mb | *D7Uwm27*  103.64 Mb | *D7Rat17^2^*  103.85 Mb | 7364936 |
| ACI.BN-(*249431173-D7Uwm28*)/Shul | Ept7.15 | *rgdv775925571-T^3^*  97.46 Mb | *rgdv775925951-C^3^*  98.51 Mb | *D7Uwm28*  101.68 Mb | *D7Uwm29*  101.75 | 13210774 |
| ACI.BN-(rs199006987-*D7Uwm27*)/Shul | Ept7.16 | *rs198543052*  101.17 Mb | *rs199006987*  101.42 Mb | *D7Uwm27*  103.64 Mb | *D7Rat17^2^*  103.85 Mb | 7364934 |
| ACI.BN-(*D7Uwm36-D7Uwm27*)/Shul | Ept7.17 | *D7Uwm35*  101.78 Mb | *D7Uwm36*  101.90 Mb | *D7Uwm27*  103.64 Mb | *D7Rat17^2^*  103.85 Mb | 7248738 |
| ACI.BN-(*D7Uwm38-D7Uwm27*)/Shul | Ept7.18 | *D7Uwm37*  101.94 Mb | *D7Uwm38*  101.99 Mb | *D7Uwm27*  103.64 Mb | *D7Rat17^2^*  103.85 Mb | 7248740 |

^1^ Rat Genome Database, <http://rgd.mcw.edu>

^2^ *D7Rat17* is annotated to two locations in Rnor_v.6.0, both on RNO7 (103.79 Mb and 103.85 Mb). See discussion for details.

^3^ These SNPs are registered in RGD and have been submitted for deposition in the European Variation Archive (study number PRJEB22833 in the European Nucleotide Archive, https://www.ebi.ac.uk/ena).
